# Survival following high-voltage electrical injury with out-of-hospital cardiac arrest: rapid ROSC and full recovery in a resource-limited setting: a case report

**DOI:** 10.1186/s12245-026-01136-x

**Published:** 2026-02-12

**Authors:** Tekiy M. Bedore, Ayto A. Negash, Amanuel D. Wakoya, Mehreteab T. Woudineh, Fitsum N. Assefa, Adey A. Bogale, Etsegenet D. Dires, Amdela M. Awoll, Mustefa M. Essa

**Affiliations:** 1 Department of Emergency Medicine and Critical Care, Werabe Comprehensive Specialized Hospital, Werabe University, Werabe, Ethiopia; 2 Department of Emergency Medicine and Critical Care, Haramaya University, Harar, Ethiopia; 3 Department of Pediatric and Child Health, St. Paul’s Hospital Millennium Medical College, Addis Ababa, Ethiopia; 4 Department of Internal Medicine, St. Paul’s Hospital Millennium Medical College, Addis Ababa, Ethiopia; 5 Department of Emergency Medicine and Critical Care, St. Paul’s Hospital Millennium Medical College, Addis Ababa, Ethiopia; 6 Department of Family Medicine, St. Paul’s Hospital Millennium Medical College, Addis Ababa, Ethiopia

**Keywords:** High-voltage electrical injury, Ventricular fibrillation, Out-of-hospital cardiac arrest, ACLS, ROSC, Resource-limited setting

## Abstract

**Background:**

High-voltage electrical injuries can cause sudden cardiac arrest, most commonly due to ventricular fibrillation, and are associated with high mortality and poor neurological outcomes. Survival with complete neurological recovery following out-of-hospital cardiac arrest remains uncommon, particularly in low-income and resource-limited settings.

**Case presentation:**

We report the case of a 22-year-old previously healthy male who sustained a high-voltage electrical injury at a construction site and collapsed immediately. On arrival at the emergency department, he was unresponsive, pulseless, and found to be in ventricular fibrillation. Cardiopulmonary resuscitation was initiated promptly, and defibrillation was performed according to advanced cardiac life support protocols, and rapid return of spontaneous circulation was achieved after three cycles of CPR and defibrillation. Post-cardiac arrest care included airway protection, close hemodynamic and neurological monitoring, oxygen supplementation, and active prevention of hyperthermia. Targeted temperature management with induced hypothermia was not performed, as the patient remained normothermic and did not develop a fever. The patient had an uneventful intensive care course and was discharged without neurological deficits.

**Conclusion:**

This case demonstrates the rare but achievable complete neurological recovery following high-voltage electrical out-of-hospital cardiac arrest presenting with a shockable rhythm, even in resource-limited settings characterized by the absence of a formal prehospital emergency medical service and limited access to advanced post-cardiac arrest interventions such as automated targeted temperature management devices. Favourable outcomes were facilitated by rapid arrival of emergency services enabling prompt rhythm identification, defibrillation with early return of spontaneous circulation, and comprehensive in-hospital post-resuscitation care, including proactive temperature control to maintain normothermia and prevent fever.

## Introduction

Electrical injuries present a unique therapeutic challenge ranging in severity from minor burns to cardiac arrest. Patients in cardiac arrest are managed following standard Advanced Cardiovascular Life Support (ACLS) guidelines [1]. The success rate of cardiopulmonary resuscitation (CPR) is much higher in this patient population [2], as they are less likely than the typical cardiac arrest patient to have underlying comorbid conditions. The European Registry of Cardiac Arrest (EuReCa) registry data indicates an average survival to hospital discharge after out-of-hospital cardiac arrest (OHCA) of approximately 7.5–8% (with wide inter-country variation, range: 3.1–35.0%), yet comparable national OHCA survival data are largely lacking for many low- and middle-income regions, including Ethiopia [3]. Survival rates for OHCA patients are higher when the collapse is witnessed, particularly with bystander CPR [4]. However, chronic conditions such as kidney disease, heart and respiratory failure, liver cirrhosis, diabetes, malignancies, and hematologic diseases significantly increase hospital mortality rates, with these comorbidities leading to up to three times the risk of fatal outcomes during hospitalization [3]. During ICU stay, 47–66% of post-OHCA patients die [5–7]. Survivors are typically younger individuals [4, 7], with a cardiac etiology for their out-of-hospital cardiac arrest (OHCA) [8]. They also tend to have a shorter duration of CPR interventions, a greater likelihood of receiving bystander CPR [8], and a higher incidence of shockable initial heart rhythms [8]. Additionally, younger age is a well-established positive prognostic factor in out-of-hospital cardiac arrest, consistently associated with higher rates of survival and favourable neurological outcomes [9].

Survival after high-voltage electrical injury complicated by out-of-hospital cardiac arrest is uncommon, and reports of rapid return of spontaneous circulation with complete neurological recovery are particularly scarce in resource-limited settings. We present this case of a 22-year-old male who suffered cardiac arrest following a high-voltage electrical burn and was discharged from the ICU after only five days in the ICU without lasting organ damage, to highlight that favorable outcomes are possible even in low-income countries when timely basic resuscitation and coordinated in-hospital care are provided.

## Case presentation

A 22-year-old previously healthy male sustained a high-voltage electrical injury while working at a construction site approximately 100 m from the hospital. Eyewitnesses reported direct contact with a high-tension electrical line via a metallic rod, resulting in immediate collapse and cardiac arrest. No cardiopulmonary resuscitation or basic life support measures were initiated. The patient was manually carried by bystanders to the Emergency Department (ED), arriving approximately 10 min after collapse. During transfer, witnesses noted slow, irregular (agonal) respirations.

On arrival at the ED, he was unresponsive, exhibiting agonal breathing and no palpable central pulse (carotid). There were no signs of effective circulation.

**Fig. 1:**
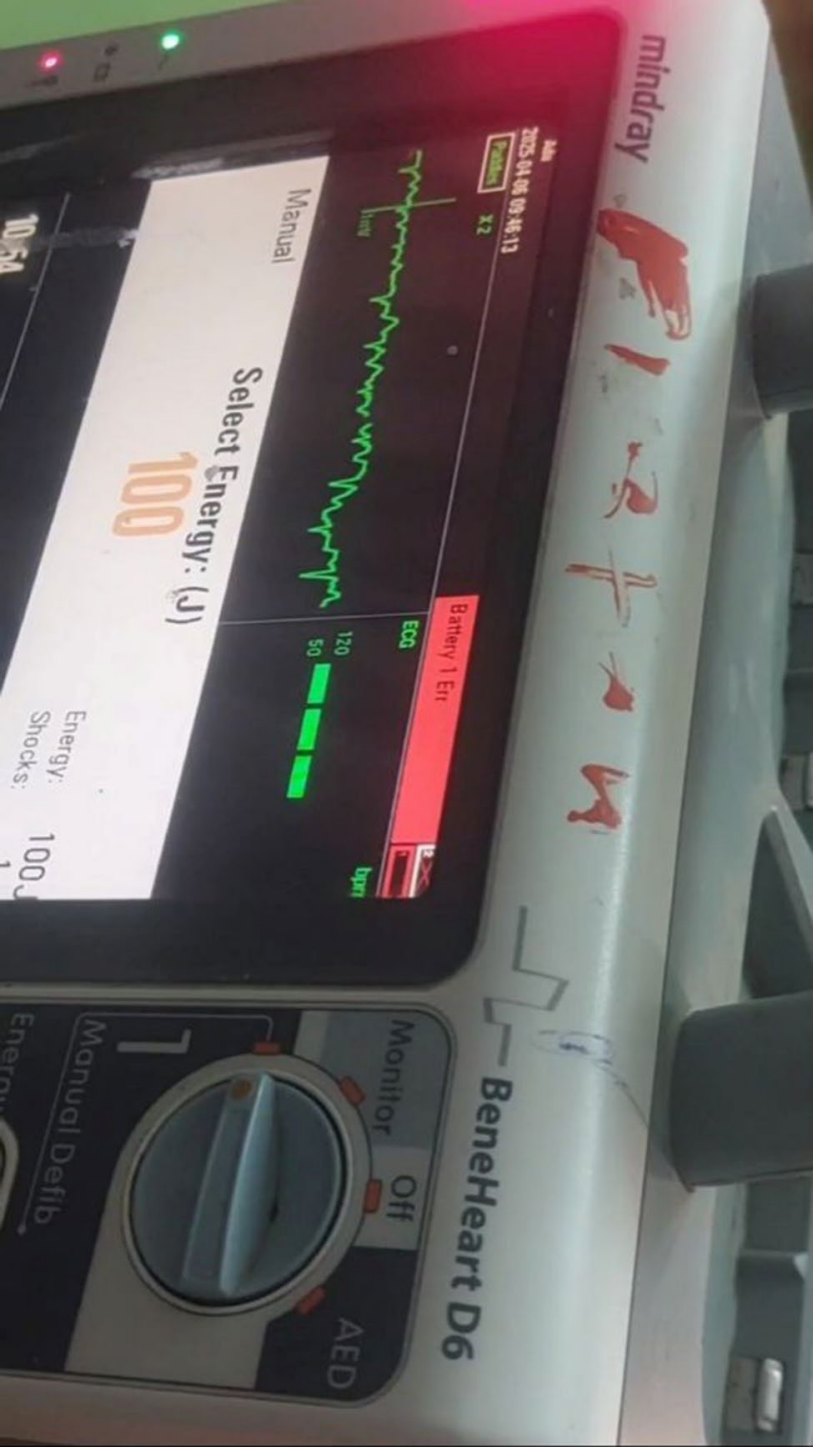
Defibrillator monitor (Mindray BeneHeart D6) displaying fine ventricular fibrillation, characterized by chaotic, low-amplitude, irregular baseline activity without identifiable QRS complexes, in manual mode

Cardiopulmonary resuscitation (CPR) was initiated immediately, and the resuscitation team was activated without delay. Defibrillator monitor revealed fine ventricular fibrillation, characterized by chaotic, low-amplitude, irregular wide QRS complex (Fig. 1). A biphasic defibrillation shock of 200 joules was delivered, followed by a full cycle of CPR. Standard advanced cardiac life support (ACLS) measures were implemented, including opening of airway with jaw thrust maneuver, and oral airway insertion and delivering 2 breaths via bag mask ventilation as protocol. Upon rhythm reassessment at the end of the second CPR cycle, VF persisted, and a second biphasic shock of 200 joules was administered. CPR was resumed promptly. During the third cycle, continued resuscitative efforts were maintained per protocol. At the end of the third cycle, rhythm check showed an organized rhythm on the monitor, and both carotid and femoral pulses were palpable, indicating return of spontaneous circulation (ROSC).

The patient was immediately intubated for airway protection and placed in the red zone of the emergency department for post-resuscitation care and close hemodynamic monitoring. Following return of spontaneous circulation, supportive post–cardiac arrest care was provided, including continuous hemodynamic and neurological monitoring, oxygen supplementation, and metabolic control. Active prevention of hyperthermia was implemented using passive strategies, including continuous core temperature monitoring, standing paracetamol 1 g PO every 6 h, and surface cooling measures during the first 24 h. The patient remained normothermic throughout the ICU course without developing fever; targeted temperature management with induced hypothermia or automated devices was not performed due to resource constraints.

A physical examination revealed electrical burn injuries with a clearly defined entry wound on the right upper extremity and an exit wound on the plantar surface of the right foot. A non-blanching, whitish wound was noted on the left thigh, involving approximately 4% of total body surface area (TBSA), consistent with a deep partial-thickness (second-degree) burn. Additionally, vesicle-forming wounds were observed on the upper chest and abdomen.

Following stabilization in the ED, two intravenous lines were secured, and blood samples were drawn for initial laboratory investigations including complete blood count, electrolytes, cardiac biomarkers, and renal function tests. Fluid resuscitation with normal saline was started. The patient was given tetanus antitoxin (3000 IU intramuscularly), and additional supportive medications were initiated including omeprazole 40 mg IV twice daily, morphine 4 mg IV QID for analgesia, and unfractionated heparin (UFH) 5000 IU subcutaneously BID.

Sedation was achieved using ketopropofol (a 1:1 mixture of ketamine and propofol) prepared by the hospital pharmacy as a standard formulation for post-cardiac arrest care. The infusion was initiated at 10 mcg/kg/min (expressed as the ketamine component) with a target Richmond Agitation-Sedation Scale (RASS) score of 0 to -2, which was successfully achieved at a maintenance rate of 30 mcg/kg/min. Additionally nasogastric tube and Foley catheter were placed.

The patient was transferred to the intensive care unit (ICU) for continued monitoring and management. He was admitted to the ICU on the same day, approximately 6 h after Emergency Department presentation. No antibiotics were administered, as there was no evidence of infection. No other adjunctive medications were given beyond those already described. He remained hemodynamically stable on mechanical ventilation. Sedation was discontinued after 24 h of normothermia maintenance. On ICU Day 2, the patient regained consciousness, becoming alert, oriented, and able to follow commands. Ventilator weaning was assessed using the ABCDE bundle approach, including Spontaneous Awakening Trials (SAT) and Spontaneous Breathing Trials (SBT via CPAP mode), which demonstrated the patient’s candidacy for liberation. He was successfully extubated on the same day without distress and subsequently weaned to room air the following morning.

Once clinically stable, the patient was transferred to the plastic surgery ward for specialized wound care and surgical management of his burn injuries. On the fifth hospital day, he underwent a split-thickness skin graft (STSG) to the left thigh. Postoperatively, he received ceftriaxone 1 g IV twice daily for 24 h, tramadol 50 mg IV three times daily.

The graft site was first opened on the eighth hospital day, showing signs of healthy adherence and no signs of infection. Emollient application was initiated as part of routine graft care. The patient continued to improve clinically, and by the fifteenth day of hospitalization, he was discharged home in stable condition, with no neurological deficits and normal vital signs.

## Discussion

High-voltage electrical injuries exceeding 1000 volts are known to cause immediate and potentially fatal arrhythmias, primarily ventricular fibrillation (VF) or asystole. These arrhythmias result from mechanisms such as direct myocardial depolarization, myocardial ischemia, or secondary hypoxia [10]. In the present case, the initial documented rhythm was VF, an immediately life-threatening but shockable arrhythmia, highlighting the critical importance of early recognition and defibrillation.

Favorable neurological outcomes following out-of-hospital cardiac arrest (OHCA) have been reported in similar cases, particularly when several key factors are present: a witnessed arrest, a shockable initial rhythm, and minimal delay in the initiation of resuscitation efforts [2, 11]. Our patient’s survival and recovery illustrate this, as basic life support (BLS) was initiated promptly, followed by rapid rhythm identification and ACLS with successful defibrillation. Early identification and management of VF in high-voltage electrocution are crucial. Literature reports, along with this case, demonstrate that even repeated defibrillation attempts can be effective in achieving ROSC when delivered promptly and according to ACLS protocols [2, 11].

Following ROSC, comprehensive post-arrest care plays a central role in determining patient outcomes. This includes airway protection, hemodynamic optimization, and intensive care unit (ICU) monitoring tailored to the electrical injury context [12].

Current post-cardiac arrest guidelines recommend proactive temperature control in all comatose adults after ROSC, irrespective of initial temperature, with the 2025 AHA advising maintenance of a constant temperature between 32 and 37.5 °C for at least 36 h and the 2025 ERC/ESICM guidelines emphasizing active prevention of fever (target ≤ 37.5 °C) for 36–72 h. Although deeper hypothermia provides no additional benefit over normothermia, fever episodes are associated with worse neurological outcomes; thus, proactive monitoring and prevention—even in initially normothermic patients—are preferred over reactive intervention alone. In this case, successful maintenance of normothermia using passive strategies (without advanced devices) aligns with these recommendations and is feasible in resource-limited settings.

In this context, a resource-limited setting refers to the absence of a formal prehospital emergency medical service, limited availability of advanced post–cardiac arrest interventions such as automated targeted temperature management devices, and reliance on bystander-initiated basic life support followed by in-hospital advanced cardiac life support and resuscitation. Despite these constraints, our patient’s complete neurological recovery highlights that timely resuscitation and adapted post-ROSC care (including passive normothermia maintenance and fever prevention) can yield excellent outcomes.

Continuous cardiac monitoring after ROSC is essential, as delayed arrhythmias have been observed in patients who initially appear stable [10]. Although the patient’s post-arrest electrocardiogram (ECG) was unremarkable, this finding should not be considered reassuring in isolation. A single normal ECG does not eliminate the risk of subsequent arrhythmogenic complications following electrical trauma [13].

Electrical injuries may also result in multi-organ dysfunction. Pulmonary complications, including direct thermal injury to lung tissue and non-cardiogenic pulmonary oedema, are well-documented in the literature [14]. In this case, meticulous ventilatory support and the absence of overt respiratory compromise may have contributed to the patient’s uneventful ICU course and timely extubation on day two.

Although the absence of bystander-initiated cardiopulmonary resuscitation represents a potential limitation, as early CPR significantly improves outcomes in out-of-hospital cardiac arrest—the estimated low-flow interval of approximately 10 min (from collapse to emergency department arrival and first defibrillation) likely contributed to the favourable neurological recovery. The presence of witnessed agonal breathing during this period is consistent with a relatively brief downtime in a shockable rhythm, where rapid professional intervention can still yield excellent results despite no prehospital BLS. This case nonetheless reinforces the importance of public education on immediate bystander CPR and access to automated external defibrillators to further optimize survival in similar scenarios.

This case demonstrates the rare but achievable complete neurological recovery following high-voltage electrical out-of-hospital cardiac arrest presenting with a shockable rhythm, even in resource-limited settings characterized by the absence of a formal prehospital emergency medical service and limited access to advanced post-cardiac arrest interventions such as automated targeted temperature management devices. Favourable outcomes were facilitated by rapid arrival of emergency services enabling prompt rhythm identification, defibrillation with early return of spontaneous circulation, and comprehensive in-hospital post-resuscitation care, including proactive temperature control to maintain normothermia and prevent fever [11, 13, 14].

## Conclusion

This case illustrates that complete neurological recovery is possible following high-voltage electrical out-of-hospital cardiac arrest with ventricular fibrillation, even in resource-limited settings lacking formal prehospital services and advanced interventions. It emphasizes the critical role of rapid professional defibrillation and adapted post-resuscitation care in achieving favourable outcomes, while highlighting opportunities for improving survival through enhanced public awareness of bystander response.

## Data Availability

No datasets were generated or analysed during the current study.

## Abbreviations

VF: Ventricular Fibrillation
ACLS: Advanced Cardiac Life Support
CPR: Cardiopulmonary resuscitation
ED: Emergency Department
ICU: Intensive Care Unit
OHCA: Out of Hospital Cardiac Arrest
IHCA: In Hospital Cardiac Arrest
ROSC: Return of Spontaneous Circulation
